# In vitro and in vivo bactericidal activity of *Tinospora sagittata (Oliv.*) *Gagnep. var. craveniana (S.Y.Hu) Lo* and its main effective component, palmatine, against porcine *Helicobacter pylori*

**DOI:** 10.1186/s12906-016-1310-y

**Published:** 2016-08-30

**Authors:** Qian Rong, Min Xu, Qi Dong, Yuli Zhang, Yinglun Li, Gang Ye, Ling Zhao

**Affiliations:** College of Veterinary Medicine, Sichuan Agricultural University, Chengdu, Sichuan 611130 People’s Republic of China

**Keywords:** Helicobacter pylori, Bactericidal activity, *Tinospora sagittata (Oliv.) Gagnep. var. craveniana (S.Y.Hu) Lo*, Palmatine

## Abstract

**Background:**

*Tinospora sagittata (Oliv.) Gagnep. var. craveniana (S.Y.Hu) Lo* (TSG) is a traditional Chinese herb that has been used for the treatment of upper respiratory tract infection and has anti-bacterial and anti-ulcer activity. Our study investigated the bactericidal effects of TSG and its major component, palmatine, against a *Helicobacter pylori* (*H. pylori*) strain isolated from pig and the standard strain *H. pylori* SS1 in vitro and in vivo.

**Methods:**

*H. pylori* was isolated from pig and named *H. pylori* SCYA201401. For in vitro experiments, the inhibitory activity of TSG and palmatine against *H. pylori* SCYA201401 and *H. pylori* SS1 were tested by use of the agar cup diffusion technique. The minimal inhibitory concentration (MIC) and minimal bactericidal concentration (MBC) were determined from the absence of *H. pylori* colonies on agar plates. Time-kill curves were used to evaluate bactericidal activity; the average number of colonies was calculated at 0 to 48 h after liquid incubation, with concentrations of drugs at 0.5, 1, and 2 × MIC. For in vivo experiments, *H. pylori* SCYA201401-infected mice were randomly divided into TSG, palmatine, triple therapy (omeprazole, clarithromycin, and amoxicillin), blank control, and model groups. The eradication ratios were determined by use of rapid urease tests and bacterial culture.

**Results:**

In vitro, the MIC and MBC of TSG against *H. pylori* SCYA201401 and SS1 were both 6250 μg/mL, whereas palmatine against *H. pylori* SCYA201401 was 6.25 μg/mL and against *H. pylori* SS1 was 3.12 μg/mL. The time-kill curves showed a dose-dependent, progressive decline in the numbers of viable bacteria up to 40 h. In vivo, the eradication ratios in the TSG and palmatine groups of mice were 80 and 50 % compared with 70 % in the triple-therapy group.

**Conclusion:**

TSG and its major component, palmatine, have bactericidal activity against *H. pylori* in vitro and in vivo*.* The possibility that TSG or palmatine can be effective in the treatment of human and animals *H. pylori* infection deserves investigation.

## Background

*Helicobacter pylori* (*H. pylori*) is a gram-negative bacterium, usually in a spiral-shaped form, which can be converted into coccoid cells under a hostile environment. *H. pylori* infection often is associated with gastrointestinal diseases, such as chronic gastritis, peptic ulcer, gastric carcinoma, and mucosa-associated lymphoid tissue lymphoma [1–7].

*H. pylori* colonizes the stomach of more than half of the world’s human population, but its mode of transmission remains unknown. Some reports indicate there is a higher prevalence of antibodies against this bacterium in veterinarians, butchers, and slaughterers than in other people, which suggests the possibility of zoonotic transmission [8–10]. Recently, investigators have isolated *H. pylori* from cows, sheep, camels, miniature pigs, and dogs’ milk. The isolated method for each animal is slightly different [11, 12].

Current treatment and eradication of *H. pylori* involve the use of triple therapy consisting of two antibiotics, inhibition of gastric acid secretion by histamine H_2_-antagonists or proton pump inhibitors, and mucosal protection provided by sucralfate and bismuth. However, the organism’s increasing antibiotic resistance and side effects arising from these drugs are creating a general health problem, indeed a global pandemic, which is especially severe in developing countries such as China [13]. Thus, safe and effective non-antibiotic agents are urgently needed. Accordingly, there is rekindled interest in the use of natural drugs, including herbs, which may possess anti-*H. pylori* activities [32] and also may have minimal side effects, easy accessibility, and affordability, even by the poor.

Traditional Chinese medicine has been used in Chinese health care for more than two thousand years. Side effects and adverse events of the traditional medicines are generally regarded as mild and infrequent [14–16]. *Tinospora sagittata* (Oliv.) *Gagnep. var. craveniana* (S.Y.Hu) *Lo* (TSG) is an important species of the genus Tinospora (Menispermaceae), a traditional Chinese Miao-nationality herb which only grows on the Mount Emei of Sichuan province, and has been widely used in the treatment of gastrointestinal diseases. TSG is listed in the Pharmacopeia of the People’s Republic of China as a plant of origin for *Tinospora sagittata* (Oliv.) *Gagnep,* with anti-inflammatory, analgesic, anti-bacterial, anti-ulcer, anti-tumor, and anti-stress activities [17–20]. The main chemical constituents of Radix Tinosporae are diterpenoid lactone, protoberberine alkaloids, aporphine alkaloids, and botanic steroids, among which diterpenoid lactones and alkaloids are considered important bioactive constituents. Palmatine is one of the major alkaloid compounds in TSG.

This study isolated a clinical *H. pylori* strain from swine by an improved method and investigated the bactericidal activities of TSG and its major component, palmatine, against the clinical *H. pylori* strain and *H. pylori* SS1 in vivo and in vitro.

## Methods

### Plant collection and extraction

TSG plants were collected from Mount Emei (Sichuan, China) between the months of September and October, 2014. The plants were identified and authenticated by Professor Qiao-jia Fan of the College of Veterinary Medicine, Sichuan Agricultural University, Chengdu, China. A voucher specimen (TSG2014) was deposited in the Veterinary Medicine Department of Sichuan Agricultural University. We prepared an extract of TSG from the plants according to reported methods [21]. Air-dried TSG (500 g) was refluxed with 95 % ethanol (*v/v*) three times each for 2 h. The solvent was completely removed in vacuo to generate a semisolid residue, which yielded 14.72 % (*w/w*) dry starting material. Stock solutions of the freeze-dried extracts were prepared for the initial screening by reconstitution in 1 % dimethyl sulfoxide (Amresco, America) to a final concentration of 1 g/mL.

### Chemicals and reagents

Palmatine, amoxicillin, omeprazole, clarithromycin, and vancomycin were purchased from Shanghai Yuanye Bio-technology, Co., Ltd (Shanghai, China). Brucella broth, Columbia blood agar, and Mueller-Hinton agar were purchased from OXOID (Hampshire, UK).

### Bacterial strain

*H. pylori* SS1 were purchased from the Chinese Center for Disease Control, Beijing.

### Sampling

One hundred seventy gastric samples were randomly selected from pigs sent to the Xingrui slaughterhouse (A29180101) in Ya’an, Sichuan Province, China, during August 28, 2014. Gastric tissue samples of 0.5 to 1 cm were obtained from the pyloric stomach immediately after slaughtering. The samples were placed in 3 mL of sterile Brucella broth with 30 % glycerol and transported rapidly to the laboratory.

### Culture and collection

The samples were homogenized in 1 mL Brucella broth with 30 % glycerol by the use of sterile mortar grinders. A 500 μL homogenate of each biopsy specimen was inoculated on selective agar plates (Columbia blood agar supplemented with 7 % fresh defibrinated sheep blood and 3 % vancomycin). Plates were incubated at 37 °C in a microaerobic atmosphere (5 % O_2_, 10 % CO_2_, 85 % N_2_) for 3 to 7 days. Isolates were presumptively identified as *H. pylori* on the basis of colony morphology (gray, small, and translucent), Gram staining, rapid urease test and biochemical reactions (oxidase, catalase, and urease positive). The bacterial colonies were collected and prepared in Brucella broth with 10 % fetal calf serum for tests.

### Identification

#### DNA extraction

Five hundred microliters of bacterium solution were centrifuged, and DNA was extracted from the precipitates with the OMEGA Bacterial DNA Kit D3350 according to the manufacturer’s protocol. The DNA extracts were eluted in a volume of 200 μL and stored in a 20 °C freezer until PCR was performed.

#### PCR assays

PCR amplification of DNA from *H. pylori* strains was performed by the use of species-specific primers (Table 1). All PCRs were performed in 25 μL volume and reaction mixtures containing 2.5 μL 10 × buffer (Mg^2+^), 2.5 μL deoxynucleotide triphosphates (dNTPs) at a final concentration of 0.25 mmol/L, 1 μL of each primer at a final concentration of 0.4 μmol/L, 0.25 μL DNA polymerase (Easy Taq, TransGen Biotech) at a final concentration of 0.05 U/μL, and 5 μL DNA extracts. PCR amplification of *H. pylori* was performed under these conditions: 3 min of pre-incubation at 94 °C, followed by 35 cycles of 20 s at 94 °C, 20 s at 60 °C, and 30 s at 72 °C. A final extension was performed for 5 min at 72 °C.

**Table 1:** Primer sequences used for *H. pylori* identification

Target Gene	Oligonucleotides of PCR primer	Products (bp)
16 s rRNA	F: CTGGAGAGACTAAGCCCTCC R: ATTACTGACGCTGATTGTGC	109
ureAB	F: AAAGAGCGTGGTTTTCATGGCG R: GGGTTTTACCGCCGCCGAATTTAA	217
cagA	F: ATAATGCTAAATTAGACAACT R: TTAGAATAATCAACAAACATC	213
vacA	F: ATGCCGCCTTTTTCACAACC R: ACGGCCCATCCACACATTAC	204

The amplification products were analyzed by the use of gel electrophoresis in 2 % agarose gels and visualized with an ultraviolet light illuminator. Nucleotide sequences were searched by use of the BLAST tool on the National Center for Biotechnology Information site (http://www.ncbi.nlm.nih.gov/ BLAST).

### Susceptibility test

Susceptibility of *H. pylori* strains to TSG and palmatine was determined by use of the agar cup diffusion technique as described [22]. Inoculated plates (Mueller-Hinton agar plates containing 5 % sheep blood) were incubated at 37 °C in an incubator under microaerophilic conditions for 3 to 7 days, after which the diameters of the zones of inhibition (mm) were measured. One percent of dimethyl sulfoxide was included in each plate as a solvent (negative) control; amoxicillin was included as positive controls.

### Minimum inhibitory concentration (MIC) and minimum bactericidal concentration (MBC) tests

Minimum inhibitory concentration (MIC) and minimum bactericidal concentration (MBC) were determined by the use of agar dilution methods according to the Clinical and Laboratory Standards Institute as described [23, 24]. Briefly, the *H. pylori* strains were inoculated on Mueller-Hinton agar plates containing 5 % sheep blood for 3 to 7 days. The bacterial colonies were collected and prepared in Brucella broth with 10 % fetal calf serum. Inoculates were prepared at a density adjusted to a 1.0 McFarland turbidity (3 × 10^8^ CFU/mL) standard. Serial dilutions (1:2) of TSG were added to the Mueller-Hinton agar for final concentrations from 200 mg/mL down to 0.39 mg/mL, and palmatine and clarithromycin (as a positive control) were added to the Mueller-Hinton agar for final concentrations from 200 μg/mL down to 0.39 μg/mL. The no-drug containing agar and 1 % dimethyl sulfoxide agar served as negative controls. Agar plates were inoculated with *H. pylori* strains and cultured for 3 days. The MIC was regarded as the lowest concentration that prevented visible growth from a duplicate experiment, and the MBC was the lowest concentration that completely inhibited bacterial growth.

### Bactericidal activities test

Bactericidal activities were evaluated by the use of time-kill curves with 0.5, 1, and 2 × MIC of drugs, and with blank, clarithromycin, and 1 % dimethyl sulfoxide controls. *H. pylori* SCYA201401 and *H. pylori* SS1 (0.1 mL at 1 × 10^8^ CFU/mL) were added to 90-mm plates with the calculated concentrations of drugs or dimethyl sulfoxide and Brucella broth with 10 % fetal calf serum (final volume 10 mL), and cultured with gentle shaking at 37 °C in a microaerobic atmosphere. At 0, 4, 8, 12, 16, 20, 24, 32, 40 and 48 h, 0.1 mL of liquid was removed, serially diluted, and plated on Columbia blood agar plates (*n* = 2 per group). Colonies were counted and averaged after 3 days of incubation [25].

### Model establishment

A total 108 mice C57BL/6 mice (6–8 week old, male/female = 1:1, weight 25 ± 5 g) were purchased from the specific pathogen-free facility at Chengdu Dossy Experimental Animals Co., Ltd. (license No. SCXK, Sichuan, 2013–24). All experimental procedures involving animals were approved by the Sichuan Agricultural University Animal Care and Use Committee (registration No. SCAU2015101801). In accordance with the Guidelines of the International Committee on Laboratory Animals, the animals were maintained in environmentally controlled rooms at 20–25 °C with a relative humidity of 55 ± 5 % and 12 h light/dark cycle. Food and water were available ad libitum. Mice were provided with sawdust bedding material and housed under these conditions for one week, then fasted for 12 h prior to the experiments.

A group of 12 mice, selected randomly as blank control, were inoculated with sterile liquid medium (Brucella broth with 10 % fetal calf serum). A group of 48 mice (the SCYA201401experimental group) was inoculated intragastrically with 0.3 mL of *H. pylori* SCYA201401 (1 × 10^8^ CFU/mL) on three alternate days. Another 48 mice (the SS1experimental group) were inoculated intragastrically with 0.3 mL of *H. pylori* SS1 (1 × 10^8^ CFU/mL) on three alternate days. Animals were fasted 24 h before and 2 h after each inoculation. After 24 days, 8 mice from the SS1 experimental group, 8 mice from the SCYA201401experimental group, and 2 mice from the control group were sacrificed by cervical dislocation without anesthesia prior to the end of the experiment, and used for assessing the by use of a rapid urea test (RUT) and bacterial culture. Only if there was a 100 % infection rate (all 18 mice positive in the RUT and bacterial culture) was the experiment continued.

### Group and drug administration

Mice of two experimental groups were randomly divided into four groups: TSG, palmatine, triple therapy (omeprazole, clarithromycin, and amoxicillin), and model groups. Drugs dosages used in in vivo experiments were equivalent to those that are used clinically. The TSG and palmatine groups were treated intragastrically with 4 g/kg and 50 mg/kg daily for 1 week, respectively. The triple therapy group was treated intragastrically with a suspension of omeprazole (0.8 μg/kg), clarithromycin (20 μg/kg), and amoxicillin (40 μg/kg) daily for 1 week [26]. The SS1 model group, the SCYA201401 model group, and blank control group were given saline solution.

### Eradication rate of *H. pylori*

At the last day of medication, all groups were deprived of feed but allowed free access to water for 24 h and then sacrificed. Stomachs were collected at the time of sacrifice, opened along the greater curvature, and washed with phosphate-buffered saline at 4 °C . Half of the antral section was isolated for RUT determination at room temperature within 24 h; the change to pink color was regarded as positive. The other half of the antral section and part of the gastric body were used for bacterial culture according to the method described above. Successful eradication of *H. pylori* was defined as negative findings from both RUT and bacterial culture.

### Statistical analysis

Eradication rates were compared among groups with a Fisher’s exact test using SPSS 20.0 software (IBM Corp., Armonk, NY, United States). *P* < 0.05 was considered statistically significant.

## Results

### Bacteria isolated and identified

Forty-two *H. pylori* strains were isolated from the 170 samples, a culture-positive rate of 24.7 %. A total of 128 biopsy samples were negative for *H. pylori* by cultivation. A clinical *H. pylori* strain, containing the gene of cagA and vacA, was named *H. pylori* SCYA201401and was used to following studies.

### Bactericidal activity in vitro

As illustrated in Table 2, the results of antimicrobial susceptibility tests (zone of inhibition, MIC, and MBC) showed that TSG and palmatine had good bactericidal activity in vitro. There was no significant different in susceptibility of *H. pylori* strains SCYA201401 and SS1. The two strains had no visible bacterial colonies after 32-h incubation on the plate containing 6250 μg/mL TSG. The *H. pylori* SCYA201401 had no visible bacterial colonies after 40-h incubation with 6.25 μg/mL palmatine, and *H. pylori* SS1 had no growth with 3.12 μg/mL palmatine. Those concentrations defined the MBCs of these agents. The results of the antimicrobial susceptibility tests revealed also that the bactericidal activity of palmatine against the *H. pylori* in vitro was significantly more than that of clarithromycin.

**Table 2:** Results of in vitro bactericidal tests

Drugs	Diameter of zones of inhibition (mm) ± SEM (100 mg)		MIC (μg/mL)		MBC (μg/mL)	
	*H. pylori* SS1	*H. pylori* SCYA201401	*H. pylori* SS1	*H. pylori* SCYA201401	*H. pylori* SS1	*H. pylori* SCYA201401
No-drugs	0	0	-	-	-	-
1 % DMSO	0	0	-	-	-	-
TSG	15.00 ± 0.12	15.48 ± 0.08	6250	6250	6250	6250
Palmatine	22.18 ± 0.00	21.24 ± 0.02	3.12	6.25	3.12	6.25
Clarithromycin	16.12 ± 0.10	23.20 ± 0.12	32	16	32	16

Notes: TSG, *Tinospora sagittata (Oliv.) Gagnep. var. craveniana (S.Y.Hu) Lo* (TSG), *DMSO* dimethyl sulfoxide

**Fig. 1:**
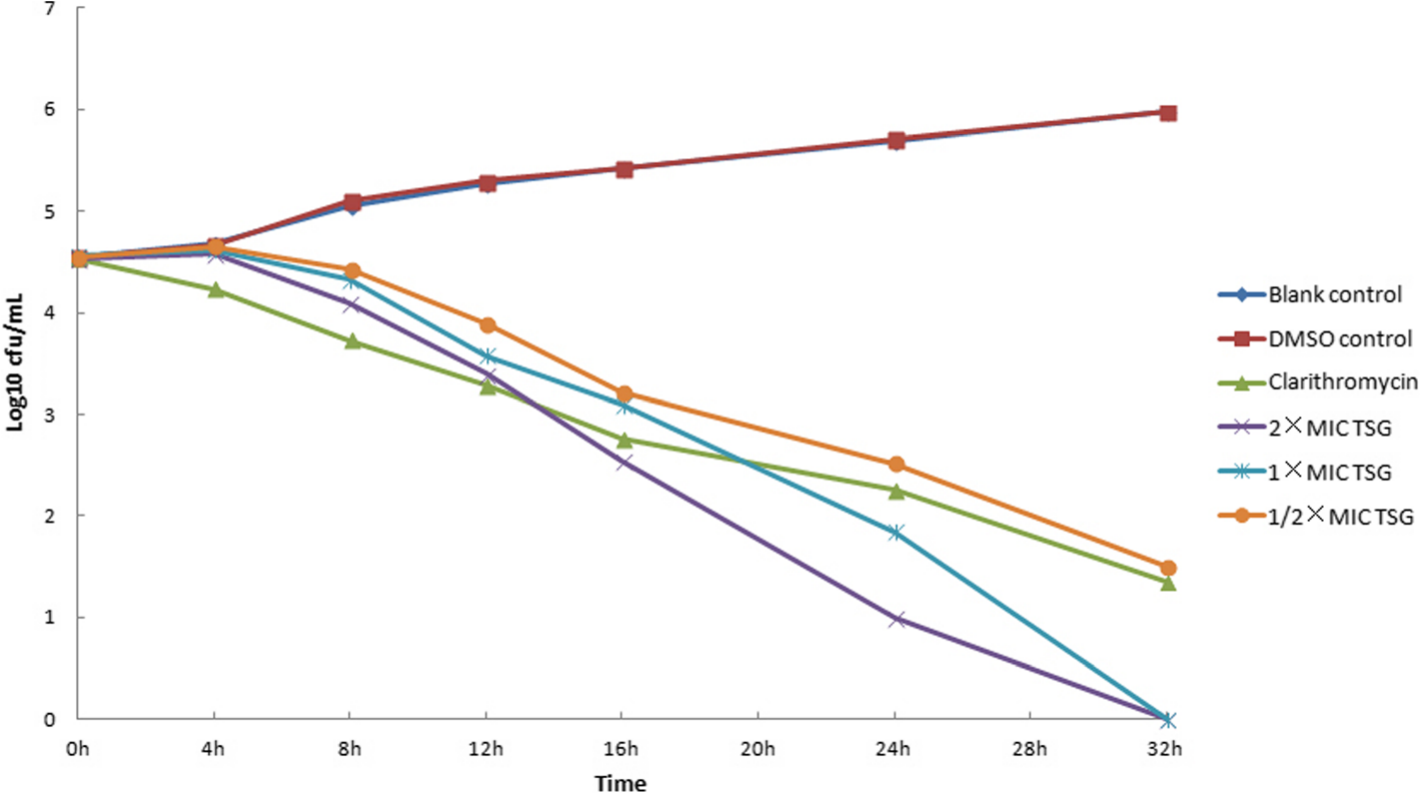
Time-kill curves of *Tinospora sagittata (Oliv.) Gagnep. var. craveniana (S.Y.Hu) Lo* against *H. pylori* SCYA201401 at different concentrations. Notes: MIC: minimum inhibitory concentration; TSG: *Tinospora sagittata (Oliv.) Gagnep. var. craveniana (S.Y.Hu) Lo*

**Fig. 2:**
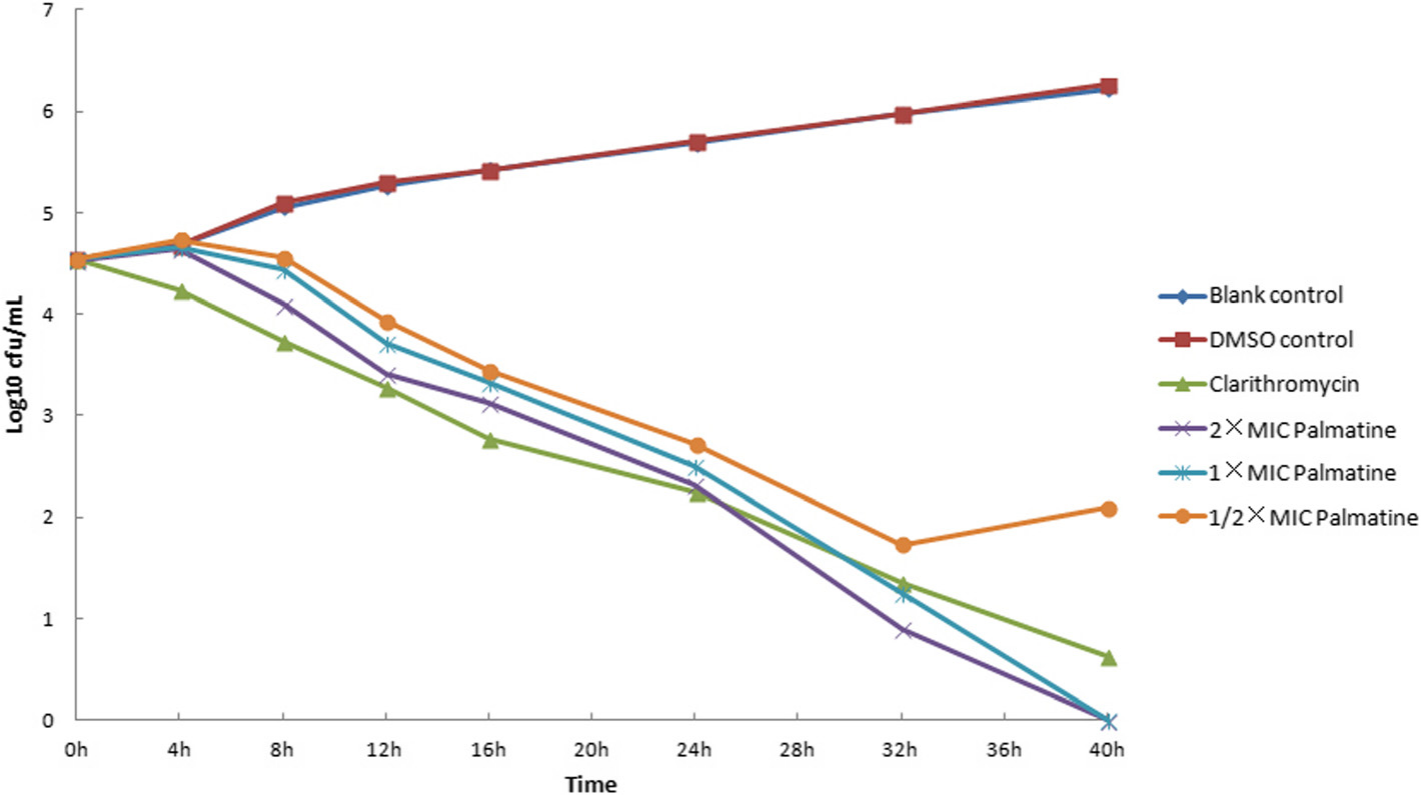
Time-kill curves of palmatine against *H. pylori* SCYA201401 at different concentrations. Note: MIC: minimum inhibitory concentration

Figures 1 and 2 show the time-kill curves of *H. pylori* SCYA201401 exposed to TSG or palmatine. The average colony count of the blank control and dimethyl sulfoxide control groups increased progressively at every time point. In contrast, the colony counts of the TSG and palmatine groups decreased steadily and did so in a dose-dependent manner. Clarithromyin similarly killed *H. pylori* SCYA201401.

### Bactericidal activity in vivo

The establishment of the *H. pylori*-infected mice model was evaluated in two ways: identification of *H. pylori* isolated from gastric tissues and the RUT. The bacteria were identified via Gram staining, RUT, and oxidase test and catalase tests. These experiments documented that a *H. pylori*-infected model was successfully established.

*H. pylori* eradication rates in the mouse model, determined with RUT and bacterial culture, are presented in Table 3. TSG was as effective as triple therapy, but palmatine was modestly less effective than those two treatments.

**Table 3:** Results of rapid urease test and bacterial culture in the mouse model (*n* = 10)

Tests	Blank control	SCYA201401experimental group				SS1 experimental group			
		Model	Triple therapy	TSG	Palmatine	Model	Triple therapy	TSG	Palmatine
rapid urease test (n)	10	0	7	9	5	0	7	9	6
Bacterial culture (n)	10	0	7	8	5	0	7	8	6
Eradication ratio	-	0/10	7/10	8/10	5/10*	0/10	7/10	8/10	6/10

*P < 0.05 vs. TSG group

## Discussion

The major goal of this study was to determine the bactericidal activities of TSG and its major component, palmatine, against *H. pylori.* The rationale underlying the goal is that the current antibiotic treatments of *H. pylori* are becoming less effective and have unwanted side effects and consequences, so effective and safer treatments, perhaps herbal medications, have been needed. In order to accomplish the study’s goal we invoked a strategy of isolating *H. pylori* from pigs, inoculating mice with the *H. pylori,* and testing the effectiveness of the traditional Chinese medication, TSG, and its principal active component, palmatine, against the isolated *H. pylori* and standard strain *H. pylori* SS1 in vitro and in vivo. The principal findings of the study are that *H. pylori* isolated from pig could readily colonize mouse stomach and that treatment of *H. pylori-*infected mice with TSG completely eliminated the *H. pylori.* Thus, the results support the notion that a traditional Chinese medication, TSG, may have therapeutic potential against human *H. pylori* infection.

TSG belongs to Tinosporae (Menispermaceae), which consists of about 34 species, with 8 species found in China. Recent studies have shown that the ethanol extract of Radix Tinosporae significantly inhibited xylene-induced ear edema and acetic acid-induced writhing in mice [20]. TSG has been used in treatment of gastritis and peptic ulcers by the Hmong for more than a thousand years. The chemical constituents of Radix Tinosporae mainly include diterpenoid lactone, protoberberine alkaloids, aporphine alkaloids, and botanic steroids, among which diterpenoid lactones (columbin) and alkaloids (columbamine, jatrorrhizine, palmatine) are considered important bioactive constituents. Phytochemical studies have shown that columbamine, jatrorrhizine, palmatine and menisperine are major diterpenoid lactones and alkaloids compounds in Radix Tinosporae [33]. Although no investigations on antibacterial activity of TSG, columbamine, jatrorrhizine and menisperine against *H. pylori* have been reported, there are a few reports on palmatine; 16 μg/mL palmatine in vitro or 50 mg/kg in vivo killed *H. pylori* [34, 35].

In our study, the MIC of TSG, 6.25 mg/mL, was sufficient to completely eliminate *H. pylori*. Although this MIC is lower than that in another study (16 μg/mL) [35], it took longer than TSG to completely eliminate the *H. pylori* strains we used. Our in vivo experiments demonstrated the potential of TSG for eradication of *H. pylori,* with efficacy equivalent to that of triple therapy. The observation that palmatine was less effective than TSG, implies that palmatine is not the only component of TSG with bactericidal effect against *H. pylori*. Like other herbs, TSG is a complex compound, and some of it effective components likely are yet unknown. Hence, further research on TSG might prove useful in identifying components that could have greater anti-*H. pylori* effect than has palmatine. Nevertheless, this study confirms the in vitro and in vivo anti-*H. pylori* effects of TSG and its main component palmatine, and provide impetus for future research with these agents.

The findings of our initial experiments with abattoir pigs have important implications. We found a substantial isolation rate (about 25 %) in abattoir pigs. Others [27] have reported that in 60 farms in England, over 79 % of porcine stomachs had either an oesophago-gastric ulcer or visible pre-ulcerative changes. Although it was not proven that *H. pylori* caused the abnormalities, it may have, or the abnormalities may have been caused by a related bacterium, such as *Helicobacter heilmanni.* The ease of transmission of porcine *H. pylori* to mice in our study indicates the possibility of zoonotic transmission of *H. pylori.* Accordingly, colonization of domestic animals has to be considered a possible cause of the high prevalence of *H. pylori* infections in man.

The current medicinal treatment of *H. pylori* is generally based on triple therapy regimen, inhibition of gastric acid secretion by histamine H_2_-antagonists, proton pump inhibitors, as well as on mucosal protective therapy provided by sucralfate and bismuth [28, 29]. Since antibiotic-resistant *H. pylori* strains triggered a global pandemic and brought various harmful adverse effects, the search for safe and effective non-antibiotic agents is urgently required [30, 31]. In recent years, active researches have rekindled interest in natural drugs possessing these activities, which are widely appreciated by the population especially in oriental countries [32].

Since improved medical therapy for human *H. pylori* infection is clearly needed, the results of this study are encouraging. Although their relevance to the management of human *H. pylori* infection is unknown, they do indicate that non-antibiotic-based treatment options, such as herbal medication, deserve consideration.

## Conclusions

In experiments with an *H. pylori* strain isolated from pigs and standard strain *H. pylori SS1,* the Chinese herbal medicine TSG and is major active component, palmatine, exhibited bactericidal effect in vitro and in a murine model. The effect was of similar magnitude to that of clarithromycin or triple therapy. In view of the increasing antibiotic resistance of *H. pylori* and the side effects associated with antibiotic-based therapy, the possibility that herbal medications could provide effective and safe therapy of *H. pylori* infection deserves investigation.

## Abbreviations

dNTPs: Deoxynucleotide triphosphates
*H. pylori*: *Helicobacter pylori*
MIC: Minimal inhibitory concentration
RUT: Rapid urease test
SS1: Sydney strain 1
TSG: *Tinospora sagittata (Oliv.) Gagnep. var. craveniana (S.Y.Hu) Lo*
